# A splicing-dependent ER retention signal regulates surface expression of the mechanosensitive TMEM63B cation channel

**DOI:** 10.1016/j.jbc.2022.102781

**Published:** 2022-12-07

**Authors:** Dan Wu, Lushan Xu, Wen-Min Cai, Shi-Yu Zhan, Guoqiang Wan, Yun Xu, Yun Stone Shi

**Affiliations:** 1 Department of Neurology, Drum Tower Hospital, Ministry of Education Key -Laboratory of Model Animal for Disease Study, Model Animal Research Center, Jiangsu Key Laboratory of Molecular Medicine, Medical School, Nanjing University, Nanjing, China; 2 Guangdong Institute of Intelligence Science and Technology, Zhuhai, China; 3 Clinical College of Traditional Chinese and Western Medicine, Nanjing University of Chinese Medicine, Nanjing, China; 4 Institute for Brain Sciences, Nanjing University, Nanjing, China

**Keywords:** TMEM63B, alternative splicing, endoplasmic reticulum retention, surface expression, mechanosensitive, osmosensitive, Co-IP, coimmunoprecipitation, ER, endoplasmic reticulum

## Abstract

TMEM63B is a mechanosensitive cation channel activated by hypoosmotic stress and mechanic stimulation. We recently reported a brain-specific alternative splicing of exon 4 in *TMEM63B*. The short variant lacking exon 4, which constitutes the major isoform in the brain, exhibits enhanced responses to hypoosmotic stimulation compared to the long isoform containing exon 4. However, the mechanisms affecting this differential response are unclear. Here, we showed that the short isoform exhibited stronger cell surface expression compared to the long variant. Using mutagenesis screening of the coding sequence of exon 4, we identified an RXR-type endoplasmic reticulum (ER) retention signal (RER). We found that this motif was responsible for binding to the COPI retrieval vesicles, such that the longer TMEM63B isoforms were more likely to be retrotranslocated to the ER than the short isoforms. In addition, we demonstrated long TMEM63Bs could form heterodimers with short isoforms and reduce their surface expression. Taken together, our findings revealed an ER retention signal in the alternative splicing domain of TMEM63B that regulates the surface expression of TMEM63B protein and channel function.

Endoplasmic reticulum (ER) retention sequence is critical in regulating the surface expression of ion channels. It serves as a quality control mechanism allowing ion channels that are correctly assembled, in which the retention signals are sterically masked, to traffic to the cell surface (1, 2, 3, 4, 5). Several ER retention signals in ion channel subunits involved in this process have been identified. The RKR signals in Kir6.1, Kir6.2, and SUR subunits of ATP-sensitive potassium channels (K_ATP_) prevent incompletely assembled channels from being exported to cell surface (2). RSRR signal in γ-aminobutyric acid B1 (GABA_B1_) subunit is masked by assembly with GABA_B2_ subunit, allowing the correctly assembled heterodimeric receptors to traffic to the plasma membrane (1). Recently, a KKK (879–881) ER retention motif was identified in GluN2A subunit of NMDA-type of glutamate receptors (NMDARs); mutation of K879R causes break of the ER retention signal, enhancement of neuronal surface/synaptic expression GluN2A-NMDARs, and impairment of synaptic plasticity and learning and memory (6). In GluN1 subunit of NMDARs, KKK and RRR motives serve as ER retention signals (7). Interestingly, the RRR ER retention signal in the C-terminal domains of GluN1 is introduced by alternative splicing (8, 9, 10, 11).

TMEM63B is an osmosensitive (or mechanosensitive) cation channel that is required for survival of outer hair cells and hearing (12, 13). Our recent study has identified a brain-specific Q/R editing at exon 20 and an alternative splicing of exon 4, resulting in four TMEM63B isoforms (QL, QS, RL, and RS respectively) (14). These two posttranscriptional procedures appear to be coupled and regulate Ca^2+^ permeability and osmosensitivity of TMEM63B channels, diversifying functional roles of TMEM63B channels in the brain. The TMEM63B homology structure model based on cryo-EM structure of OSCA1.2 indicated that the Q/R editing site is located at the intracellular opening of the channel pore (14, 15, 16). The positively charged arginine residue at Q/R site reduces the Ca^2+^ permeability of the channel (14). The exon 4 encodes amino acid sequence (aa 80–93) located at the intracellular loop-1 (aa 63–157) between transmembrane helices M0 and M1. The inclusion of exon 4 reduces the osmosensitivity of TMEM63B but does not affect the Ca^2+^ permeability (14). How exon 4 alternative splicing regulates the channel osmosensitivity remains unclear.

Sequence analysis suggested that the 14 amino acids encoded by exon 4 might contain two putative RXR-type ER retention signals (RLRR 80–83 and RER 86–88). Indeed, we found that the long TMEM63B isoform containing exon 4 exhibited reduced surface expression compared to the short variant. Mutagenesis analysis suggested that the RER motif served as a functional ER retention signal, mutation of which enhanced surface expression of the long TMEM63B (TMEM63B-QL). More long form TMEM63B proteins bound to the retrieval COPI vesicles than the short ones further verifying that the RER motif is an ER retention signal of TMEM63B. In addition, heterodimers could form by long and short TMEM63Bs, in which the long TMEM63B negatively regulated the trafficking of the short isoform. Our study thus revealed a novel splicing-dependent ER retention signal that regulates surface trafficking and function of TMEM63B channels.

## Results

### TMEM63B splicing variants show different surface expression pattern

**Figure 1:**
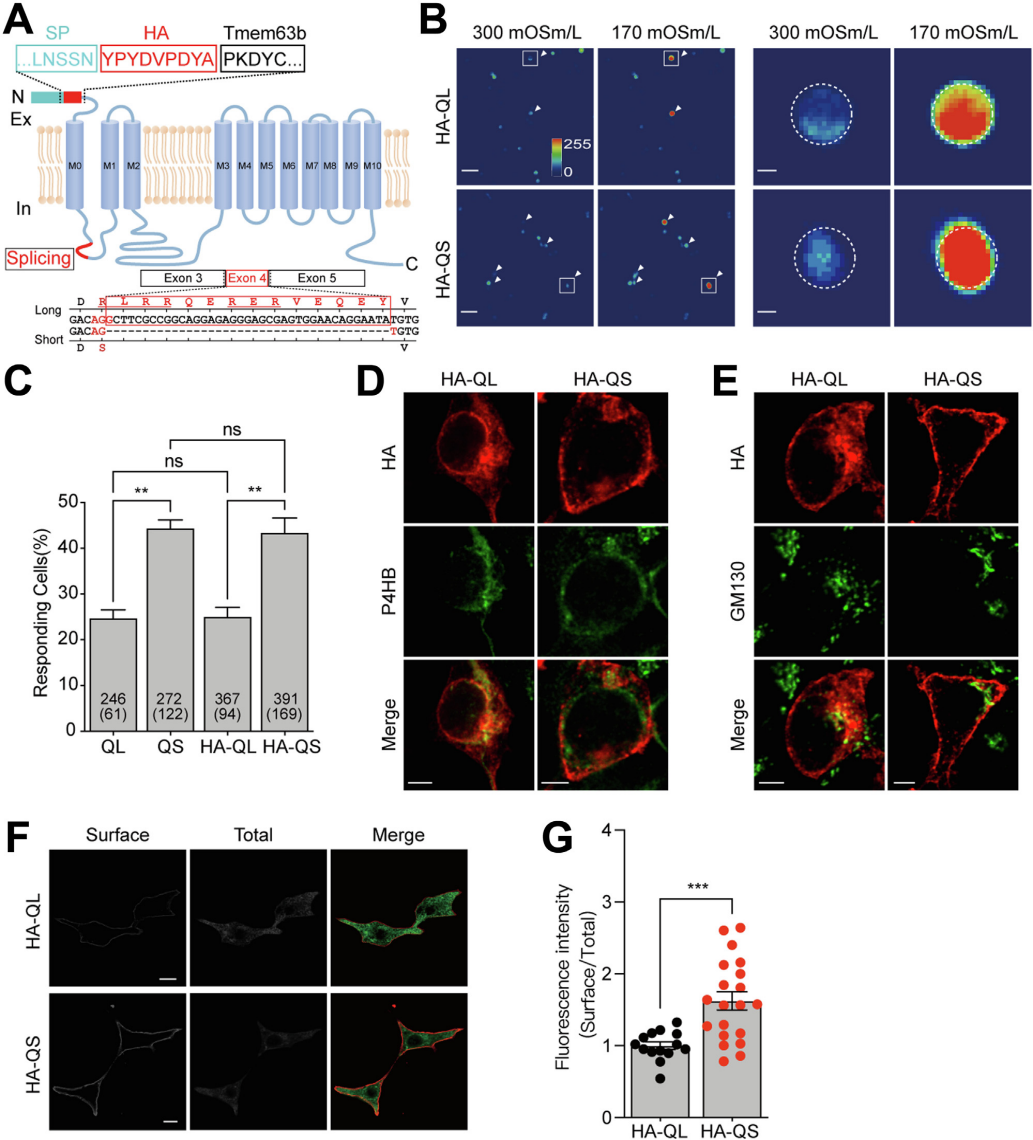
**TMEM63B splicing variants show different surface expression capability.***A*, topology structure of the mouse TMEM63B splicing variants. The nucleotides and corresponding amino acid sequences of alternative splicing motif are shown under the structure. SP, signal peptide. *B*, fluorescent emission images of N2a cells transfected with HA-TMEM63B-QL or HA-TMEM63B-QS, accompanied with the calcium indicator GCaMP6f, in response to 170 mOsm/liter hypotonic stimuli. Left panel, The scale bar represents 50 μm. Right panel, the scaled-up images of the box marked in left panel. The dotted circles indicated the cell boundaries. The scale bar represents 5 μm. *C*, percentage of cells responding to 170 mOsm/liter hypotonic solutions. Numbers of cells tested and responding (in parentheses) are indicated in the bars. QL (24.6 ± 1.7%, n = 3), QS (44.1 ± 1.8%, n = 3), HA-QL (25.0 ± 2.1%, n = 3), HA-QS (43.3 ± 3.2%, n = 3). Data are shown as mean ± SEM. ∗∗*p* < 0.01; *ns*, not significant; one-way ANOVA followed by Tukey's multiple comparisons test. *D* and *E*, colocalization of HA-TMEM63B-QL/QS with ER protein P4HB and Golgi protein GM130 by the permeabilized immunostaining in HEK293T cells 48 h after transfection. The scale bar represents 5 μm. *F*, immunofluorescence staining of the surface and total TMEM63B in QL and QS variants. The scale bar represents 10 μm. *G*, quantification of the ratios of surface relative to total TMEM63B in HA-QL and HA-QS variants. The ratios were normalized to that of HA-QL. HA-QL (1.000 ± 0.053, n = 14), HA-QS (1.624 ± 0.126, n = 20). Data are shown as mean ± SEM. ∗∗∗*p* < 0.001; unpaired two-tailed *t* test. ER, endoplasmic reticulum.

To examine the trafficking of TMEM63B, we constructed HA epitope tagged TMEM63B and expressed HA-TMEM63B with (QL) or without exon 4 (QS) in HEK293T cells. The HA-tag was inserted after the N-terminal signal peptide (https://services.healthtech.dtu.dk/service.php?SignalP), thus HA should be located extracellularly when TMEM63B was delivered to the cell membrane (Fig. 1*A*). The surface HA was detected by anti-HA antibody under living condition, while TMEM63B was detected by an antibody against C-terminal sequence of TMEM63B only when cells were permeabilized (Fig. S1*A*), indicating the topology of extracellular N terminus and intracellular C terminus (Fig. 1*A*). Untransfected HEK293T cells was not stained by anti-TMEM63B antibody, indicating that endogenous TMEM63B, if present, was low (Fig. S1*B*). In addition, equal amounts of total proteins were detected in QL, QS, HA-QL, and HA-QS transfected cells (Fig. S1*C*), indicating HA-tag does not affect TMEM63B expression. We then examined whether the functional properties of TMEM63B isoforms have been altered by the HA-tag. Previously, we have established that the osmosensitivity of TMEM63 family proteins is reliably represented by the responding rate of transfected N2a cells to hypoosmotic pressure (12, 14). We thus expressed the TMEM63B with or without HA tag accompanied with the calcium indicator GCaMP6f in N2a cells. GCaMP6f fluorescence was monitored after switching the extracellular osmolarity from 300 mOsm/liter to 170 mOsm/liter. Consistent with previous results (14), the Ca^2+^ fluorescence elevation occurred more frequently in cells expressing QS/HA-QS than those expressing QL/HA-QL (Fig. 1, *B* and *C*). HA insertion did not change channel osmosensitivity.

The permeabilized immunostaining indicated that TMEM63B proteins were widely distributed on cell surface and intracellularly (Fig. S1*B*). Quite amount of them were colocalized with ER protein P4HB (Fig. S2*A*) and the Golgi protein GM130 (Fig. S2*B*) after transfection for ∼20 h, suggesting the channels are synthesized through classic ER-Golgi pathway. HA immunostaining at 48 h after transfection showed that HA-TMEM63B-QL had apparently more intracellular contents than HA-TMEM63B-QS (Fig. 1, *D* and *E*), indicating weaker trafficking capability of long form TMEM63B than short isoform. The surface expression of HA-TMEM63B was quantified by the ratio of surface protein intensity relative to total proteins using a two-step immunostaining method (6, 17). The surface HA-TMEM63B was detected by a mouse anti-HA antibody under membrane impermeable condition. Then, the cells were permeabilized to examine the total HA-TMEM63B using a rabbit anti-TMEM63B antibody recognizing the intracellular C-terminal sequence. We found that the surface expression of HA-TMEM63B-QL was relatively lower than HA-TMEM63B-QS (Fig. 1, *F* and *G*), indicating that the amino acid sequence encoded by exon 4 may contain molecular signals regulating the surface expression of TMEM63B.

### An RER motif in the alternative splicing sequence restricts the surface expression of TMEM63B

**Figure 2:**
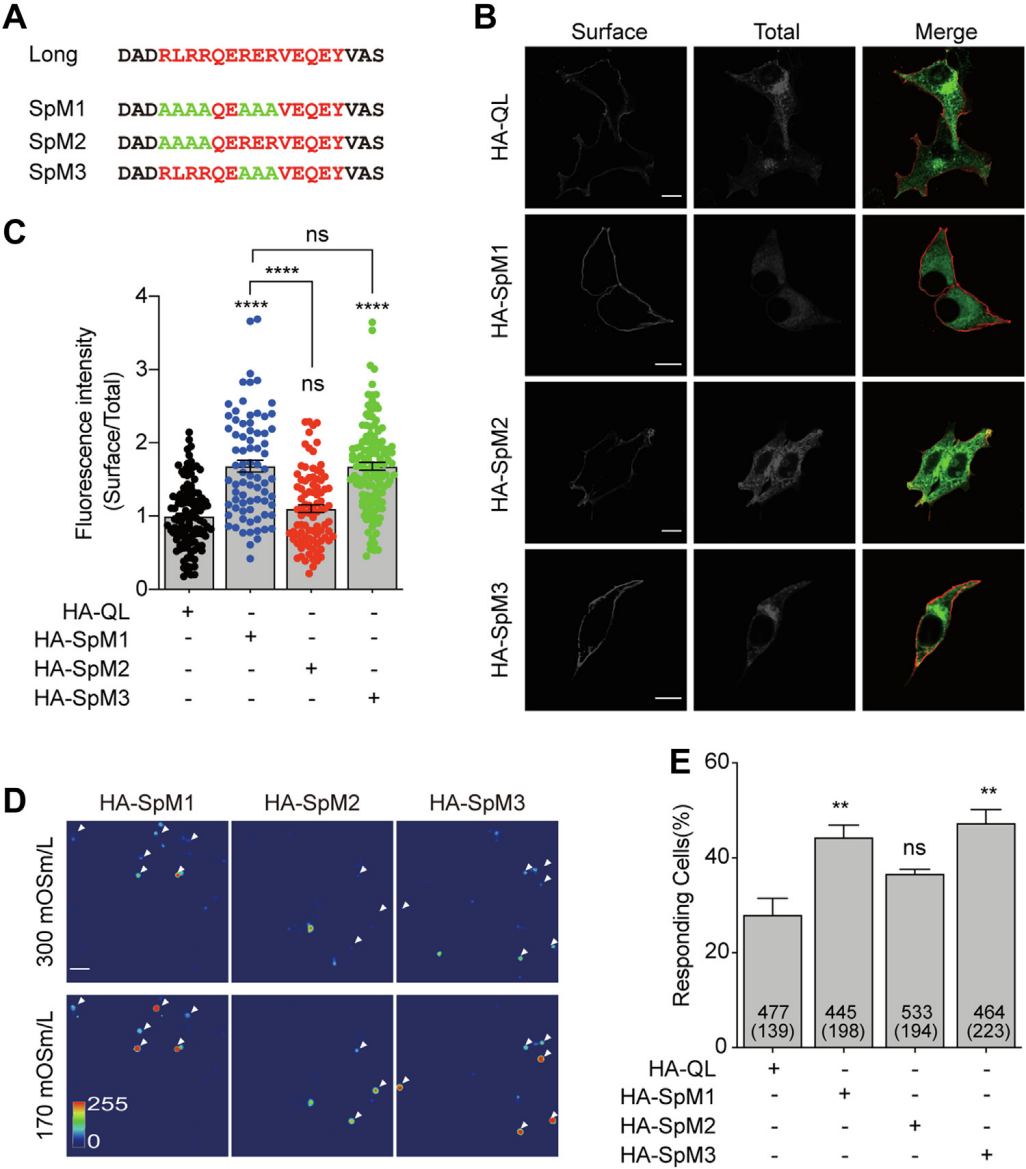
**The segment encoded by exon 4 restricts the surface expression of TMEM63B.***A*, mutations of the putative ER retention signals in exon 4 encoded segment. *B*, immunostaining of the surface and total TMEM63B in the corresponding mutants shown in (*A*). The scale bar represents 10 μm. *C*, quantification of the ratios of surface relative to total TMEM63B in the corresponding mutants shown in (*A*). The ratios were normalized to that of HA-QL. HA-QL (1.000 ± 0.041, n = 114), HA-SpM1 (1.683 ± 0.080, n = 77), HA-SpM2 (1.103 ± 0.053, n = 91), HA-SpM3 (1.681 ± 0.052, n = 130). *D*, fluorescent emission images of N2a cells transfected with ER retention signal related mutants, accompanied with the calcium-sensitive reporter GCaMP6f, in response to 170 mOsm/liter hypotonic stimuli. The scale bar represents 50 μm. *E*, percentage of cells responding to 170 mOsm/liter hypotonic solutions. Numbers of cells examined and responding (in parentheses) are indicated in the bars. HA-QL (28.5 ± 3.8%, n = 3), HA-SpM1 (44.2 ± 2.5%, n = 3), HA-SpM2 (36.4 ± 0.8%, n = 3), HA-SpM3 (47.4 ± 2.7%, n = 3). Data are shown as mean ± SEM. ∗∗*p* < 0.01; ∗∗∗∗*p* < 0.0001; *ns*, not significant; one-way ANOVA followed by Tukey's multiple comparisons test. ER, endoplasmic reticulum.

The exon 4 is 39 nucleotides long, which takes part in encoding 14 amino acids. Compared to TMEM63B-QS, TMEM63B-QL contains 13 additional residues and a preceding arginine residue that is serine in TMEM63B-QS (Fig. 1*A*). These 14 residues contain two putative RXR-type ER retention signals, (RLRR and RER, Fig. 1*A*). We mutated these motives to alanine residues combinedly (SpM1) or individually (SpM2 and SpM3, respectively) in HA-TMEM63B-QL and evaluated the surface expression in HEK293T cells (Fig. 2*A*). Mutating both of RLRR and RER sequences (SpM1) resulted in significantly increased expression of surface TMEM63B. The enhanced surface expression was also detected in the RER mutant (SpM3) but not in the RLRR mutant (SpM2, Fig. 2, *B* and *C*), indicating that the RER motif is likely an ER retention signal restraining the surface expression of TMEM63B. In addition, hypoosmotic stress induced Ca^2+^ influx occurred more frequently in N2a cells expressing SpM1 or SpM3 relative to the WT HA-TMEM63B-QL, while cells expressing SpM2 showed no significant enhancement in osmosensitivity (Fig. 2, *D* and *E*). Thus, the osmosensitivity of TMEM63B mutants were consistent with their surface expression pattern.

### RER motif is an ER retention signal

**Figure 3:**
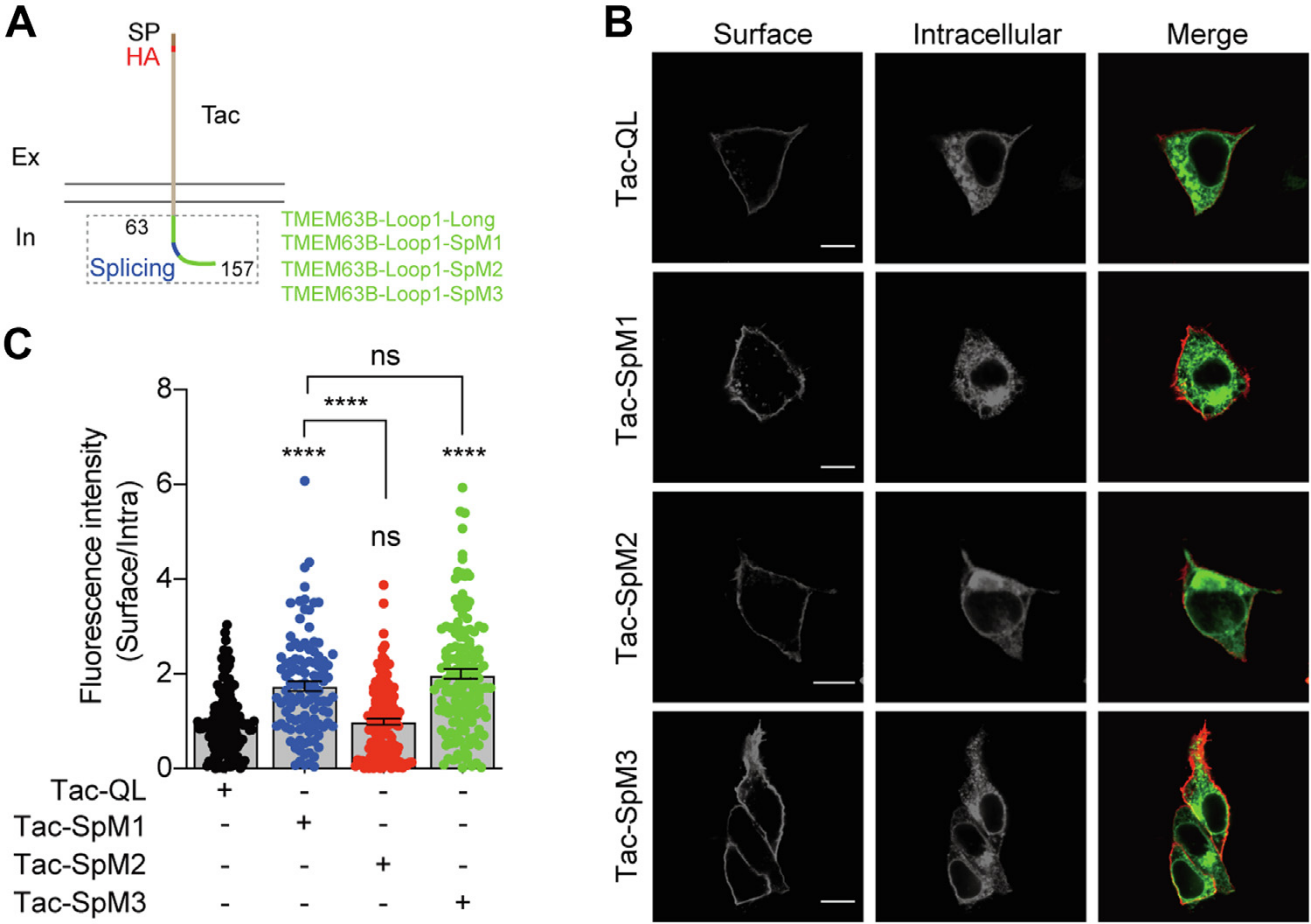
**The RER motif restricts the surface expression of chimeric Tac.***A*, the chimeric constructs of Tac and TMEM63B intracellular loop1 (amino acids 63∼157, TMEM63B-loop1). HA-tag was inserted after the N-terminal signal peptide for immunostaining detection. *B*, immunostaining of the surface and intracellular Tac chimeras. The scale bar represents 10 μm. *C*, quantification of the ratios of surface relative to intracellular chimeric Tac. The ratios were normalized to that of Tac-TMEM63B-loop1-long chimera. Tac-QL (1.000 ± 0.055, n = 151), Tac-SpM1 (1.741 ± 0.103, n = 107), Tac-SpM2 (0.991 ± 0.066, n = 137), Tac-SpM3 (1.972 ± 0.100, n = 149). Data are shown as mean ± SEM. ∗∗∗∗*p* < 0.0001; *ns*, not significant; one-way ANOVA followed by Tukey's multiple comparisons test.

To further verify if the RER is an ER retention signal, we examined its regulation on the human interleukin-2 receptor subunit alpha (Tac), a type-I transmembrane protein containing a short intracellular C-terminal tail that is natively expressed on the cell surface (11). The TMEM63B intracellular loop1 was fused to HA-tagged Tac (Fig. 3*A*). The surface and intracellular proteins of Tac-Tmem63b-loop1 chimeras were evaluated by the HA signals under intact and permeabilized conditions. Mutating both of RLRR and RER sequences (Tac-SpM1) significantly enhanced surface HA-signal compared to the WT chimeras. The increment of membrane HA signal was also detected in RER mutant (Tac-SpM3) but not in RLRR mutant (Tac-SpM2, Fig. 3, *B* and *C*). These results further verified that the RER motif in long form TMEM63B is an ER retention signal.

### Long form TMEM63B is more volunteer to bind COPI retravel complex

**Figure 4:**
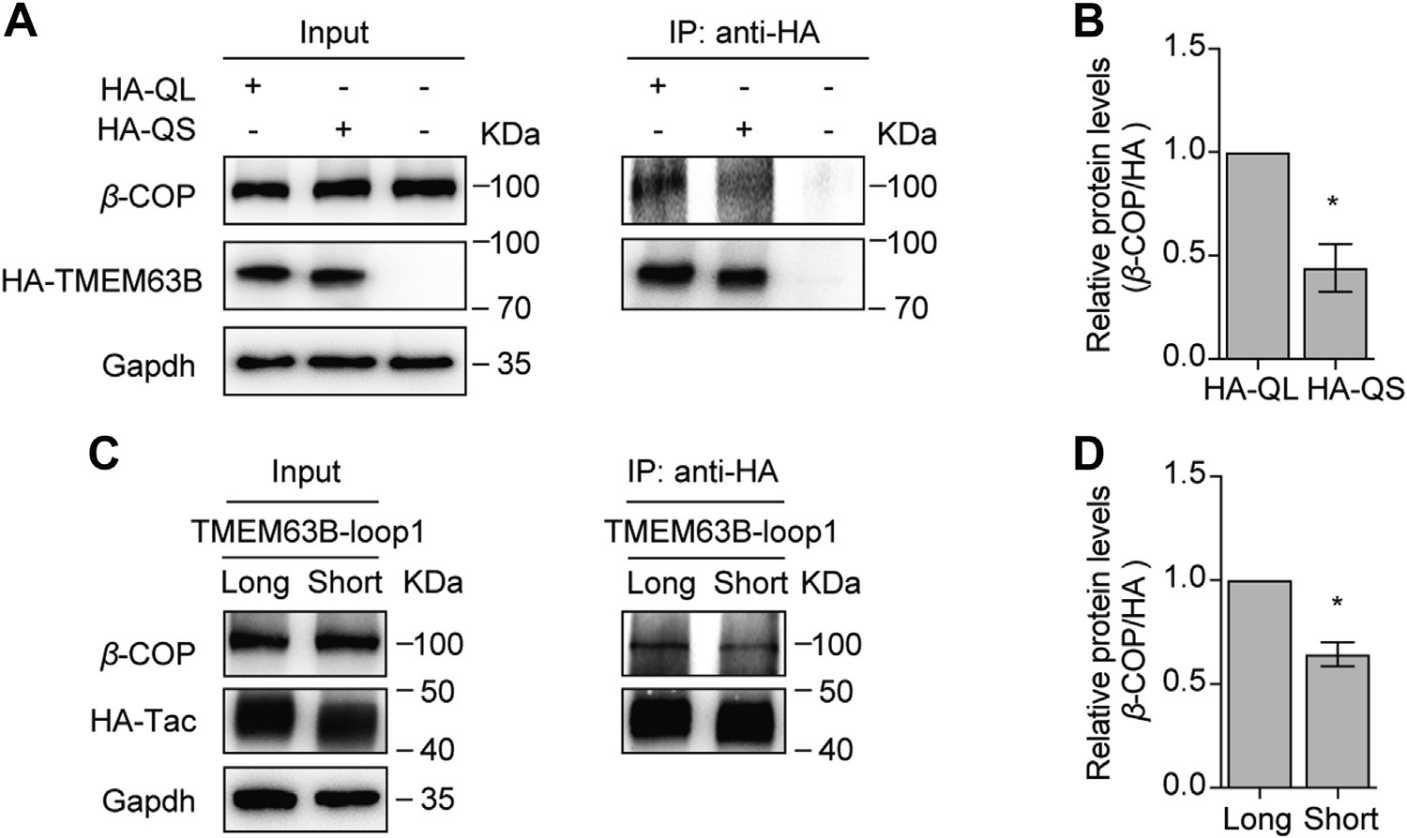
**More *β*-COP proteins interacted with TMEM63B-QL than TMEM63B-QS.***A* and *B*, the interaction between HA-TMEM63B-QL/QS and *β*-COP subunit of COPI complex. *C* and *D*, the interaction between Tac-TMEM63B-loop1 chimeras and *β*-COP subunit. Data are shown as mean ± SEM. ∗*p* < 0.05; paired two-tailed *t* test.

Proteins on the plasma membrane are synthesized in ER and traffic to cell membrane *via* Golgi apparatus. Some membrane receptors or ion channels are retrieved from Golgi apparatus and transported back to ER (retrograde transport) through coatomer protein I (COPI) retrieval mechanism. Several studies demonstrated that RXR motif is responsible for binding to COPI complex in Golgi compartment (18, 19, 20, 21). If the RER motif in the TMEM63B serves the same role, long TMEM63B would bind to COPI complex more than short one. Indeed, more *β*-COP proteins were precipitated by HA-TMEM63B-QL than HA-TMEM63B-QS (Fig. 4, *A* and *B*). Similar results were obtained in the Tac-TMEM63B-loop1 chimeras (Fig. 4, *C* and *D*). Taken together, these data indicated that more long form TMEM63B proteins are retrieved by COPI vesicles and transported to ER retrogradely than short form proteins, plausibly explained the lower surface expression level of long TMEM63B.

### TMEM63B-QL negatively regulates TMEM63B-QS trafficking

**Figure 5:**
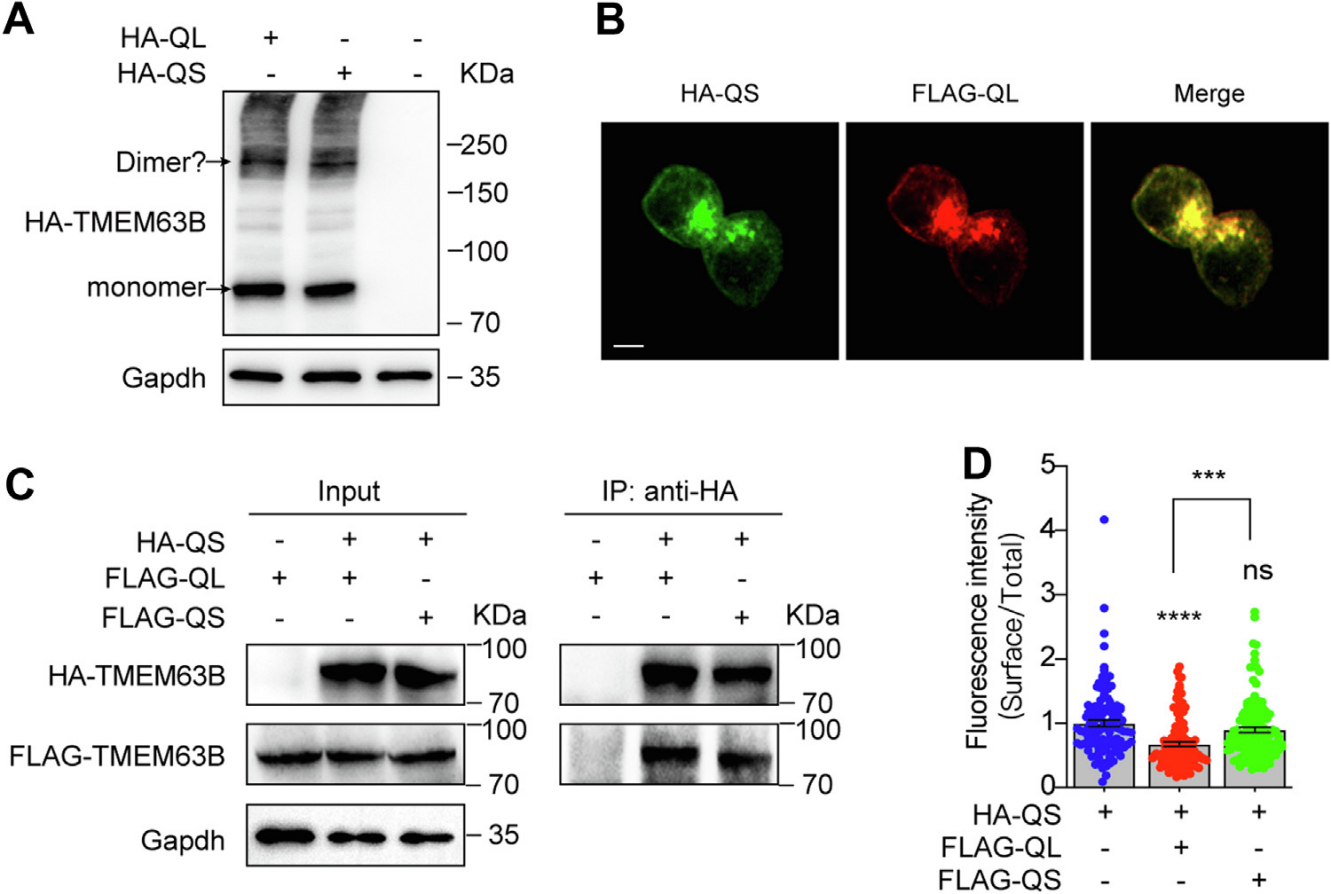
**The long form TMEM63B negatively regulates surface trafficking of short TMEM63B.***A*, putative dimer bands of HA-TMEM63B-QL and HA-TMEM63B-QS. *B*, the colocalization of HA-TMEM63B-QS and FLAG-TMEM63B-QL in HEK293T cells. The scale bar represents 5 μm. *C*, the interaction of FLAG-TMEM63B-QL/QS with HA-TMEM63B-QS. *D*, quantification of the ratios of surface relative to total TMEM63B in HA-QS, HA-QS + FLAG-QL, and HA-QS + FLAG-QS expressing cells. The ratios were normalized to that of HA-TMEM63B-QS. HA-QS (1.000 ± 0.052, n = 111), HA-QS + FLAG-QL (0.675 ± 0.034, n = 128), HA-QS + FLAG-QS (0.898 ± 0.043, n = 126). Data are shown as mean ± SEM. ∗∗∗*p* < 0.001; ∗∗∗∗*p* < 0.0001; *ns*, not significant; one-way ANOVA followed by Tukey's multiple comparisons test.

The TMEM63B homology structure model based on cryo-EM structure of OSCA1.2 indicated that the functional channels are symmetric homodimers (14, 15, 16). Western analysis of HA-TMEM63B-QL and HA-TMEM63B-QS presented a large molecular weight band ∼200 KDa in addition to ∼90 KDa monomer band (Fig. 5*A*). The larger band likely represented putative TMEM63B dimers. We wondered whether the long and short TMEM63Bs can form heterodimers. FLAG-TMEM63B-QL/QS were constructed, in which the FLAG epitope was inserted at the same position as HA-tag in HA-TMEM63Bs, and coexpressed with HA-TMEM63B-QS. When HA-QS and FLAG-QL were coexpressed, colocalization of HA and FLAG signals indicated similar expression and trafficking patterns of long and short variants (Fig. 5*B*). The coimmunoprecipitation (Co-IP) assay showed that HA-QS efficiently pulled down FLAG-QS (Fig. 5*C*), suggesting tagged HA and FLAG epitopes did not interrupt TMEM63B-QS dimerization. Interestingly, HA-QS pulled down FLAG-QL with the same efficiency, indicating that heterodimers were formed between TMEM63B-QL and TMEM63B-QS (Fig. 5*C*). We then examined the surface expression of the heterodimers. As expected, coexpression of FLAG-QS did not affect surface/total ratio of HA-QS. However, coexpression of FLAG-QL reduced the surface expression of HA-QS (Fig. 5*D*). These data suggested that long TMEM63B negatively regulates surface expression of the short form.

## Discussion

In this study, we identified an RER motif in the alternative splicing sequence encoded by exon 4 of TMEM63B as an ER retention signal. The long isoforms of TMEM63B containing this additional RER motif are more susceptible to associate with COPI retravel vesicles and retrogradely transported back to ER from Golgi apparatus. The RER ER retention signal thus restricts the functional channel proteins on cell surface, providing a plausible mechanistic explanation for the reduced osmosensitivity of the long TMEM63B splicing variants (14).

How does the RER motif regulate protein retention in ER? The homology structural model for TMEM63B based on the cryo-EM structure of OSCA1.2 indicated that the functional TMEM63B channels are symmetric homodimers (14, 15, 16). One possibility is that the dimerization of TMEM63B may sterically mask the RER ER retention signals. Thus, the RER ER retention signal in TMEM63B may act as a quality control mechanism that allows the correctly assembled dimeric channels to traffic to the cell surface. Similar theory has been proposed in several ion channels. The RKR signals in Kir6.1, Kir6.2, and SUR subunits of ATP-sensitive potassium channels (K_ATP_) and the RSRR signal in γ-aminobutyric acid B1 (GABA_B1_) subunit prevent incompletely assembled channels from being exported to cells surface (1, 2). It would be of interest to know if TMEM63B monomers can traffic to cell surface. The RER motif in the first intracellular loop of TMEM63B is mediated by inclusion of exon 4. This type of ER retention signal also occurs in the C-terminal of NMDAR GluN1 subunit, where the RXR signal is introduced by alternative splicing, allowing isoform-specific regulation on the surface expression of NMDA receptors (8, 9, 10, 11). Thus, alternative splicing may play critical roles in the assembly of some ion channels.

Previously, we have analyzed the splicing of *TMEM63B* in several tissues. In most tissues, exon 4 was included in the mature transcript of *TMEM63B*. A minor portion of *TMEM63B* in heart excludes exon 4. However, the majority of *TMEM63B* (∼80%) in the brain excludes exon 4 (14). The TMEM63B in the brain may thus have stronger surface expression than in other tissues. We speculate this may accommodate with the specific neuronal requirements. As variable ion channels are abundantly expressed in neurons, substantial amount of membrane TMEM63B may be required to manifest its regulatory effects, although such effects remain to be illustrated. Interestingly, the short TMEM63B in the brain is mostly edited at the Q/R site, resulting in a charged arginine residue (Arg619) at the inner opening of TMEM63B channel pore, leading to a reduction in Ca^2+^ permeability (14). The enhanced surface expression and reduced Ca^2+^ permeability indicates that the putative TMEM63B function in the brain relies more on currents but not Ca^2+^ entrance.

Our previous results suggested that there are four different types of TMEM63B subunits in the brain, that is, RS, QL, QS, and RL, with variable expression levels (14). Here, we showed that the long and short TMEM63Bs could freely assemble to form heterodimers when coexpressed in HEK293T cells. This would further expand the diversity of the functional TMEM63B channels in the central nervous systems, assuming the rule is applicable in the brain. Interestingly, the surface trafficking of TMEM63B-QS was downregulated when coexpressed with TMEM63B-QL, indicating the long isoforms of TMEM63Bs might play a regulatory role for the short isoforms, the majority of TMEM63Bs in the brain. Future work is required to illustrate the physiological function of TMEM63B variants in the brain as well as in other tissues.

## Experimental procedures

### Molecular biology

The complementary DNAs of long and short *TMEM63B* splicing variants were generated in our previous study (12, 14). The *TMEM63B* mutants and HA/FLAG-tagged recombinants were constructed through overlapping PCR and specific primers (Table S1) and then subcloned into pCDNA3.1 vectors by Ligation-Free Cloning Kit (abm, E001). The coding sequence of Tac were amplified from human activated T lymphocytes and subcloned into pCAGGS vectors. The HA-tagged chimeras, with the TMEM63B intracellular loop1 (amino acids 63∼157) fused to the C-terminal of Tac, were generated by overlapping PCR and corresponding primers (Table S1). For measuring the cytoplasmic calcium changes, the free calcium indicator GCaMP6f was fused to the C-terminal of TMEM63B through a P2A linker (TMEM63B-P2A-GCaMP6f), leading to separate expression of TMEM63B and GCaMP6f (12, 14).

### Immunofluorescence

The surface expression was quantified by the ratio of surface proteins intensities relative to total proteins using a two-step immunostaining method as previously reported (6, 17). In brief, HEK293T cells that seeded on poly-D-Lysine–coated coverslip were fixed with 4% PFA for 10 min on ice at 20∼24 h after transfections. After blocking in 5% goat serum, the surface proteins were labeled with a mouse anti-HA primary antibody (Sigma–Aldrich, H3663, 1:500) at room temperature (RT) for 2 h, followed by Alexa-555 secondary antibody (Invitrogen, A-21422, 1:1000). Then, the cells were permeabilized with blocking serum containing 0.3% TritonX-100. Next, for TMEM63B splicing variants and mutants, the total TMEM63B was immunostained with a customized rabbit anti-TMEM63B primary antibody (abcam, 1:200) that recognize a peptide located in intracellular C-terminal, followed by Alexa-488 secondary antibody (Invitrogen, A32731, 1:1000). For Tac chimeras, the intracellular proteins were immunostained with a rabbit anti-HA antibody (Cell signaling technology, H3724, 1:250), followed by Alexa-488 secondary antibody. Images were obtained by confocal microscope (ZEISS, LSM880) and analyzed with ImageJ software (National institutes of Health). The maximum projection of Z-stack images was used for integrated intensity quantification. Each group of cells used for comparison was imaged with the same acquisition parameters.

For permeabilized immunostaining, if not specified, cells were fixed at ∼20 h after transfection and then permeabilized with blocking serum containing 0.3% TritonX-100 and then immunostained with the corresponding primary antibodies: anti-HA (Sigma–Aldrich, H3663, 1:500), anti-HA (Cell signaling technology, H3724, 1:250), anti-FLAG (Sigma–Aldrich, F1804, 1:500), anti-TMEM63B (abcam, 1:200), anti-P4HB (ABclonal, A0692, 1:50), and anti-GM130 (ABclonal, A16248, 1:200), followed by the Alexa-conjugated secondary antibody. For live-cell labeling, the surface HA-TMEM63B were labeled by incubating the live HEK293T cells with anti-HA antibody (Sigma–Aldrich, H3663, 1:500) diluted in prewarmed culture medium at 37 °C for 20 min. Followed by washing, cells were incubated with secondary antibody at RT for 20 min and then fixed with 2% PFA. After fixation, cells were subjected to permeabilized immunostaining.

### Cytoplasmic Ca^2+^ measurements

The cytoplasmic Ca^2+^ influx was monitored by free calcium indicator GCaMP6f as previously reported (12, 14). TMEM63B-P2A-GCaMP6f vectors were transfected into N2a cells mounted on the coverslip. Forty hours after transfection, the cells were perfused with isotonic extracellular solution (in mM): 70 NaCl, 5 KCl, 1 CaCl_2_, 1 MgCl_2_, 10 Hepes, and 10 glucose (pH 7.4 adjusted with NaOH; 300 mOsm/liter adjusted with mannitol). The isotonic solution was exchanged to 170 mOsm/liter hypotonic solution without changing the ionic concentrations by a peristaltic pump (Longer Precision Pump, BT100-2J, China) at a constant speed. The osmolarity was measured by a vapor pressure osmometer (Wescor, Vapro 5600). The cytoplasmic calcium fluorescence was recorded at 1 Hz for 10 min by the Hamamatsu digital imaging camera (Hamamatsu, C11440-22U) at RT (24 ± 2 °C) using 488 nm illumination. The change of fluorescence was normalized by the ratio of real-time intensity (Ft) relative to the initial value (F0). The cells with Ft/F0 > 1.5 were considered as positive responses to hypotonic challenge.

### Western blots

HEK293T cells were lysed in radioimmunoprecipitation buffer (EpiZyme, PC103) with 1 mM PMSF solution and a mixture of protease inhibitors (Roche), at about 30 h after transfection. Lysates were kept on ice for 30 min, followed by centrifugation for 30 min at 12,000 rpm at 4 °C. Then, the supernatants were mixed with 5× loading buffer (Mei5bio, MF145-01) before being separated by 6% SDS-PAGE gels (Beyotime, P0050A). Proteins were immunoblotted onto polyvinylidene difluoride membranes (Millipore), which were blocked in 5% nonfat milk dissolved in 150 mM NaCl, 10 mM Tris-Base (pH 7.4), and 0.1% Tween 20 at RT for 1 h. The membranes were then incubated with anti-HA (Cell signaling technology, H3724, 1:1000), anti-FLAG (Sigma–Aldrich, F7425, 1:1000), anti-TMEM63B (abcam, 1:1000), anti-*β*-COP (abcam, ab2899, 1:1000), anti-Gapdh (Bioworld, MB001, 1:10,000) primary antibodies, and horseradish peroxidase–conjugated secondary antibodies (Bioworld). The reactive protein bands were developed by ECL kit (Tanon, 180–501) and captured by a chemiluminescent imaging system (Tanon). To quantify protein expression, the integrated absorbances of protein bands were measured using ImageJ software (National Institutes of Health).

### Co-IP assays

HEK293T cells were lysed as described in Western blot experiments. The supernatants centrifugated from the cell lysates were incubated with anti-HA antibody (Sigma–Aldrich, H3663, 1:125) at 4 °C overnight and then incubated with Protein G agarose beads (Invitrogen, 10004D) at 4 °C for 2 h on a rotating platform. After incubation, beads were washed five times with lysis buffer. For the Co-IP assays between HA-TMEM63B and *β*-COP, HA-TMEM63B, and FLAG-TMEM63B, the washed beads were incubated with 0.1 M glycine at pH 2.5 at RT for 30 min. The eluates were then subjected to Western blot analysis. For the Co-IP assays between Tac chimeras and *β*-COP, the washed beads were incubated with 5× loading buffer and boiled at 95 °C for 5 min before being subjected to Western blot analysis. For all samples, 1% of that used for immunoprecipitations was used for input.

### Statistical analysis

All data are presented as means ± SEM. Statistical analyses were performed using the GraphPad Prism software (version 8.0) (GraphPad Software Inc) and analyzed using one-way ANOVA, followed by Tukey's multiple comparisons test, paired or unpaired *t* test, if not otherwise stated. *p* values less than 0.05 were considered statistically significant. ∗*p* < 0.05; ∗∗*p* < 0.01; ∗∗∗*p* < 0.001; ∗∗∗∗*p* < 0.0001. *p* ≥ 0.05 was denoted as “*ns.*”

## Data availability

All data supporting our conclusions are contained within this article and in the supporting information.

## Supporting information

This article contains supporting information.

## Conflict of interest

The authors declare that they have no conflicts of interest with the contents of this article.
