# Supplementary material for: A splicing-dependent ER retention signal regulates surface expression of the mechanosensitive TMEM63B cation channel
Source: J Biol Chem. 2022 Dec 7;299(1):102781. doi: 10.1016/j.jbc.2022.102781 (PMC9830214; doi:10.1016/j.jbc.2022.102781)
Supplement: Supporting information [file mmc1.pdf]

Supporting information

A splicing-dependent ER retention signal regulates surface expression of the mechanosensitive TMEM63B cation channel

Dan Wu, Lushan Xu, Wen-Min Cai, Shi-Yu Zhan, Guoqiang Wan, Yun Xu, Yun Stone Shi

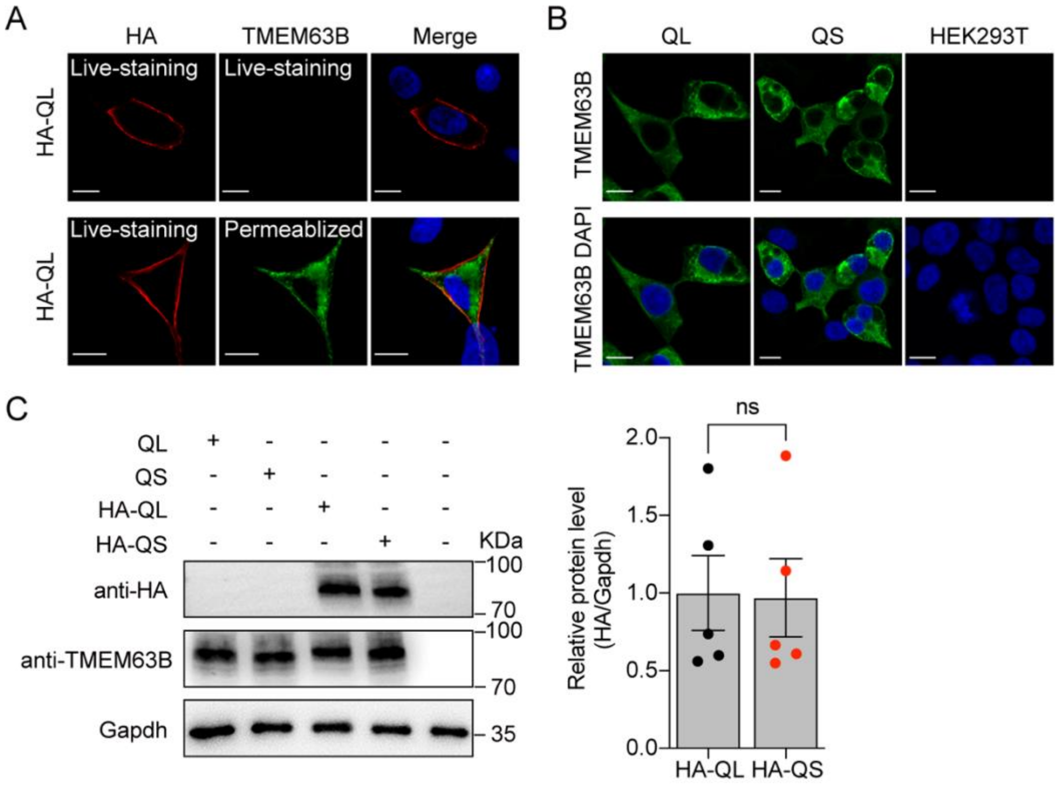

Supplementary Figure 1. The immunostaining and western blot results of QL, QS, HA-QL and HA-QS in HEK293T cells. A. The living-staining of surface HA followed by immunostaining with TMEM63B under permeabilization. Scale bar, 10 μm. B. The immunofluorescence results of HEK293T cells expressing TMEM63B-QL and TMEM63B-QS. HEK293T without transfection showed no clear expression of endogenous TMEM63B. Scale bar, 10 μm. C. Left panel: the western blot results of the cell expressing QL, QS, HA-QL and HA-QS. Right panel: Quantification of the expression levels of HA-QL and HA-QS. Data are shown as mean ± SEM. *ns*, not significant; unpaired two-tailed *t* test.

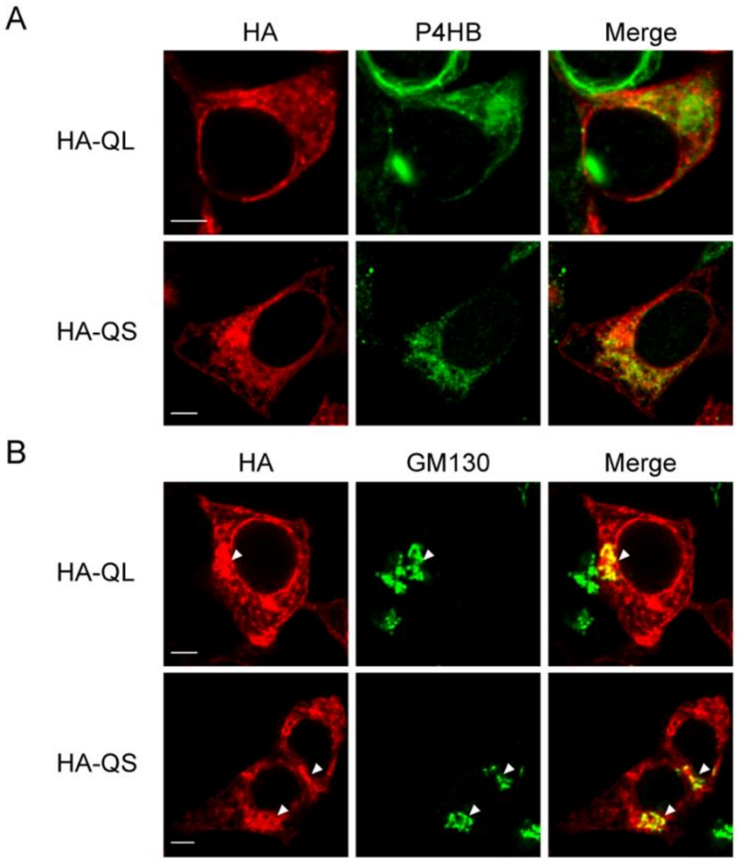

Supplementary Figure 2. The subcellular location of HA-TMEM63B in HEK293T cells. Co-localization of HA-TMEM63B-QL/QS with ER protein P4HB (A), Golgi protein GM130 (B). Cells were transfected for ~20 hours. Scale bar, 5 µm.

Table S1. Primers for expression constructs in this study

Primer	Sequence (5'-3')
TMEM63B-F	ATAGGGAGACCCAAGCTTGCCACCATGCTGCCGTTCTTGCTG
TMEM63B-R	AGCTCCGCTTCCGATATCCTGGTGAATCTCATTCTC
TMEM63B-HA-F	ACGATGTACCTGACTATGCAAAGGACTACTGCTACAGTG
TMEM63B-HA-R	TCAGGTACATCGTATGGGTACGGGTTGCTGCTGTTGAG
TMEM63B-FLAG-F	CAAAGACGATGACGACAAGAAGGACTACTGCTACAGTG
TMEM63B-FLAG-R	TCGTCATCGTCTTTGTAGTCCGGGTTGCTGCTGTTGAG
TMEM63B-SpM1-F	CAGGAGGCTGCAGCTGTGGAACAGGAATATGTGGCATCAGCTATGCATG
TMEM63B-SpM1-R	CACAGCTGCAGCCTCCTGTGCAGCTGCTGCGTCTGCATCTGTCACCAAG
TMEM63B-SpM2-F	GCAGCAGCTGCACAGGAGAGGGAGCGAG
TMEM63B-SpM2-R	CTGTGCAGCTGCTGCGTCTGCATCTGTCACCAAG
TMEM63B-SpM3-F	AGGCTGCAGCTGTGGAACAGGAATATGTGGCATC
TMEM3B-SpM3-R	TTCCACAGCTGCAGCCTCCTGCCGGCGAAGCCTG
hTAC-F	GAATTCGCGGCCGCTAGCGCCACCATGGATTCATACCTGCTG
hTAC-R	AATTCGAAGCTTGAGCTCGAGCTAGATTGTTCTACGCGTTC
hTAC-HA-F	TACGATGTTCCAGATTACGCTGGAGGAGGAGGATCAGAGCTCTGTGACGATGAC
hTAC-HA-R	ATCTGGAACATCGTATGGGTATGATCCTCCTCCTCCTGCCTGGCAGCCAGGCAC
hTAC-loop1-F	GAGGAAGAGTAGAACGCGTCGGAAGGTGGCCTGGGAC
hTAC-loop1-R	GAAGCTTGAGCTCGAGTTAGTGTCTTTGGAAGGACAG
